# Cerebral oxygen extraction and blood flow in community-based older adults: associations with white matter hyperintensity and neurocognitive function

**DOI:** 10.1093/braincomms/fcag056

**Published:** 2026-02-24

**Authors:** Yifan Yan, Xuhao Zhao, Yaping Zhang, Wanxin Li, Zhiying Lin, Yi Zhou, Shenghao Fang, Jingkai Huang, Christopher Li-Hsian Chen, Zixuan Lin, Xin Xu

**Affiliations:** Department of Psychiatry, School of Public Health, The 2nd Affiliated Hospital of Zhejiang University School of Medicine, Hangzhou 310058, China; Department of Brain-Computer Interface, Nanhu Brain-Computer Interface Institute, Hangzhou 311100, China; The State Key Lab of Brain-Machine Intelligence, MOE Frontier Science Center for Brain Science and Brain-Machine Integration, Zhejiang University School of Medicine, Hangzhou 310058, China; Key Laboratory of Intelligent Preventive Medicine of Zhejiang Province, Hangzhou, Zhejiang 310058, China; Department of Psychiatry, School of Public Health, The 2nd Affiliated Hospital of Zhejiang University School of Medicine, Hangzhou 310058, China; Key Laboratory of Intelligent Preventive Medicine of Zhejiang Province, Hangzhou, Zhejiang 310058, China; Department of Psychiatry, School of Public Health, The 2nd Affiliated Hospital of Zhejiang University School of Medicine, Hangzhou 310058, China; Key Laboratory of Intelligent Preventive Medicine of Zhejiang Province, Hangzhou, Zhejiang 310058, China; Department of Psychiatry, School of Public Health, The 2nd Affiliated Hospital of Zhejiang University School of Medicine, Hangzhou 310058, China; Key Laboratory of Intelligent Preventive Medicine of Zhejiang Province, Hangzhou, Zhejiang 310058, China; Department of Psychiatry, School of Public Health, The 2nd Affiliated Hospital of Zhejiang University School of Medicine, Hangzhou 310058, China; Key Laboratory of Intelligent Preventive Medicine of Zhejiang Province, Hangzhou, Zhejiang 310058, China; Department of Psychiatry, School of Public Health, The 2nd Affiliated Hospital of Zhejiang University School of Medicine, Hangzhou 310058, China; Key Laboratory of Intelligent Preventive Medicine of Zhejiang Province, Hangzhou, Zhejiang 310058, China; Department of Psychiatry, School of Public Health, The 2nd Affiliated Hospital of Zhejiang University School of Medicine, Hangzhou 310058, China; Key Laboratory of Intelligent Preventive Medicine of Zhejiang Province, Hangzhou, Zhejiang 310058, China; Department of Psychiatry, School of Public Health, The 2nd Affiliated Hospital of Zhejiang University School of Medicine, Hangzhou 310058, China; Key Laboratory of Intelligent Preventive Medicine of Zhejiang Province, Hangzhou, Zhejiang 310058, China; Memory, Ageing, and Cognition Centre (MACC), Department of Pharmacology, Yong Loo Lin School of Medicine, National University of Singapore, Singapore 119077, Singapore; 6 Key Laboratory for Biomedical Engineering of Ministry of Education, Department of Biomedical Engineering, College of Biomedical Engineering & Instrument Science, Zhejiang University, Hangzhou, Zhejiang 310027, China; Department of Psychiatry, School of Public Health, The 2nd Affiliated Hospital of Zhejiang University School of Medicine, Hangzhou 310058, China; Department of Brain-Computer Interface, Nanhu Brain-Computer Interface Institute, Hangzhou 311100, China; The State Key Lab of Brain-Machine Intelligence, MOE Frontier Science Center for Brain Science and Brain-Machine Integration, Zhejiang University School of Medicine, Hangzhou 310058, China

**Keywords:** small vessel disease, white matter hyperintensity, cognitive decline, cerebral oxygen extraction fraction, cerebral blood flow

## Abstract

Cerebral oxygen extraction fraction (OEF) and cerebral blood flow (CBF) are key haemodynamic markers. Emerging evidence suggests that they may exert compensatory effects on small vessel disease and cognitive outcomes, with potentially nonlinear relationships, particularly in community-dwelling seniors. Therefore, we conducted a cross-sectional study of 296 participants from the Heritage Study in China. OEF was assessed using T2-relaxation-under-spin-tagging (TRUST) MRI, while CBF was measured using phase contrast MRI. White matter hyperintensity (WMH) volumes were segmented through T2 fluid-attenuated inversion recovery (FLAIR) imaging and log-transformed. Neurocognitive function was evaluated across multiple domains and summarized as a global composite *Z*-score. Based on the median values of CBF and OEF, participants were categorized into four quadrants and generalized linear models were used to examine associations between OEF CBF patterns and WMH and cognition. Participants with high OEF and low CBF had highest WMH volume (4.48 ± 8.02 cm3) and worse cognitive performance (−0.13 ± 1.04). Overall, higher OEF was significantly related to lower global cognition (*P* = 0.012), whereas lower CBF was significantly associated with greater WMH burden (*P* = 0.001). Compared with those in high OEF and low CBF, individuals in low OEF and high CBF exhibited significantly lower WMH volume (*β* = −0.55, 95% confidence interval (CI) = [−1.05, −0.05]) and better cognition (β = 0.28, 95% CI = [0.02, 0.54]). In contrast, low OEF and low CBF were associated with relative cognitive reserve (*β* = 0.32, 95% CI = [0.02, 0.61]) but higher WMH volume. Domain-based analyses for attention, visuospatial and memory functions showed similar results. To further explore potential nonlinear effects, response surface analysis was performed to investigate relationships among OEF, CBF, WMH, and global cognition, revealing a significant association between CBF and WMH (*β* = −1.42, 95% CI = [−2.85, −0.01]). For global cognitive performance, OEF was negatively associated with cognitive outcomes (OEF: *β* = −0.49, 95% CI = [−0.87, −0.11], OEF²: *β* = 0.01, 95% CI = [0.00, 0.01]), indicating a U-shaped association between OEF and cognition. Notably, when CBF was high, cognition was relatively preserved even under higher OEF. In summary, OEF emerged as a sensitive marker of cognitive vulnerability in community-based seniors, particularly in attention, executive function, visuospatial ability, and memory, while CBF was the primary determinant of WMH burden. Combined OEF CBF patterns enabled classification of at-risk community-dwelling individuals, with the ‘misery perfusion’ pattern (high OEF, low CBF) showing the most adverse profile and representing a promising target for early risk stratification.

## Teaser

Yan *et al*. report that in community-dwelling older adults, higher cerebral oxygen extraction with lower cerebral blood flow (‘misery perfusion’) is associated with greater white matter hyperintensity burden and poorer cognitive performance, whereas better matching of blood supply to oxygen use relates to healthier brain structure and neurocognitive function.

## Graphical Abstract

**Graphical Abstract:**
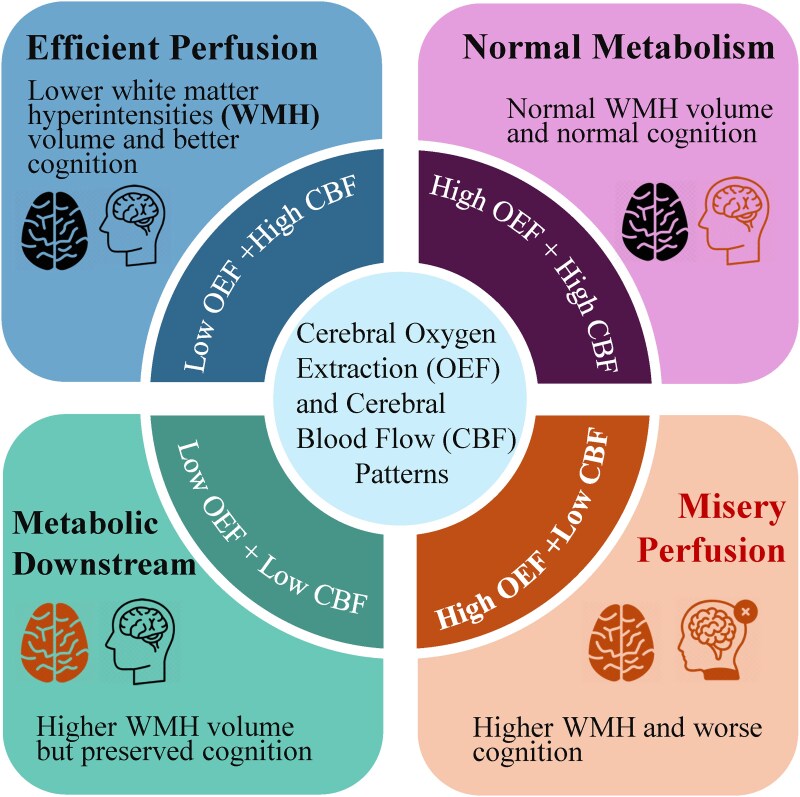

## Introduction

Cerebral oxygen extraction fraction (OEF) and cerebral blood flow (CBF) are microvascular markers indicative of early vascular dysfunction, as blood vessels play a critical role in delivering oxygen and nutrients to the brain.^1^ OEF reflects the brain's ability to extract oxygen from circulating blood and serves as an indicator of cerebral oxygen utilization.^2,3^ Novel neuroimaging biomarkers, T2-relaxation-under-spin-tagging (TRUST), allow for rapid and non-invasive evaluation of cerebral haemodynamics.^4^ OEF tends to increase with advancing age^5^; higher OEF has been reported as a predictor of cerebrovascular disease.^6,7^ This reflects a state in which cerebral blood supply is insufficient relative to metabolic demand, requiring the brain to extract a greater fraction of oxygen from the incoming blood.^3^ Moreover, OEF could offer valuable insights into the cognitive impairment,^3,8^ yet prior findings are potentially modulated by different pathology, and have rarely been validated in large community cohorts or mapped onto domain-specific cognition.

CBF represents the vascular system’s capacity to deliver oxygen and glucose to metabolically active brain regions, and is typically maintained through autoregulatory mechanisms.^9^ In a healthy brain, CBF and metabolic demand are tightly coupled, resulting in a stable OEF.^5^ However, impaired perfusion can disrupt this balance and has been associated with white matter damage, reduced cognitive reserve and increased risk of dementia, even in the absence of overt cerebrovascular events.^10,11^ ‘Misery perfusion’, defined as reduced CBF with a compensatory increase in OEF, is considered a hallmark of potentially reversible cerebral ischaemia,^12^ typically seen under chronic haemodynamic stress such as carotid occlusion, and often preceding stroke or transient ischaemic symptoms.^13,14^ PET-MRI studies provided further evidence of such compensatory increases in OEF in elderly individuals associated with age-related cerebrovascular disease and lower cognitive scores.^15^ Although prior studies indicate that ‘misery perfusion’ is a risk state for large-vessel pathology, its relevance to cerebral small vessel disease (SVD) remains insufficiently defined, especially in community-based populations.

Emerging evidence also suggests that the relationships among OEF, CBF and small vessel disease may be nonlinear. A recent article reported that chronic hypoperfusion is a key contributor to SVD, and that OEF may follow an ‘increase-then-decrease’ trajectory with disease progression.^16^ White matter hyperintensities (WMH), the most typical imaging marker of SVD, are thought to result from oxygen stress, chronic hypoperfusion and impaired cerebrovascular reactivity.^17,18^ Building on this theoretical and empirical foundation, our study extends the investigation to a large community-based memory cohort, considering OEF and CBF together to characterize haemodynamic patterns, highlighting potential opportunities for clinical risk stratification. Inspired by recent applications of response surface analysis in psychological research,^19,20^ we modelled OEF and CBF as interacting physiological indices and test their nonlinear associations with WMH burden and cognition.

To address these gaps, this study aims to: (i) characterize the distinct haemodynamic patterns of OEF and CBF, and determine whether the combination of high OEF and low CBF (‘misery perfusion’) is associated with greater WMH burden and poorer cognitive performance compared with other quadrants; (ii) model the continuous and potentially nonlinear relationships of OEF and CBF with WMH and cognition to elucidate the interplay between cerebral oxygen utilization, perfusion, vascular burden and cognitive outcomes in community-based older adults.

## Materials and methods

### Study participants

This study was conducted within the Chinese Study for Prevention dementia among Community-Dwelling Older Adults (the Heritage Study^21^), a community-based cohort of older adults in Hangzhou, designed to investigate cerebrovascular health and cognitive decline. Participants were recruited through a multistage stratified sampling strategy. From February 2023 to July 2024, 296 eligible community-dwelling older participants aged 50 (middle-life and old age) and above were recruited in Gongshu District, Hangzhou, China. The sex distribution of the analytic sample was as follows: 92 of 296 participants were male (31.08%) and 204 were female (68.92%), yielding a male-to-female ratio of 1:2.22. Participants underwent comprehensive neuropsychological testing, neuroimaging, physical examinations and clinical investigations. This study was performed in accordance with the Declaration of Helsinki. Ethical approval was obtained from Zhejiang University.

Inclusion Criteria: Age 50 years or older; community-dwelling individuals; capable of completing MRI and cognitive assessments; provided written informed consent.

Exclusion Criteria: Diagnosis of acute stage stroke and transient ischaemic symptoms; diagnosis of dementia; history of major psychiatric disorders (e.g. schizophrenia, major depressive disorder requiring hospitalization); contraindications to MRI (e.g. pacemaker, metallic implants, severe claustrophobia); poor image quality due to motion artefacts or technical issues; evidence of significant brain pathology on MRI (e.g. large infarcts, tumours).

### Cognitive assessments

All participants completed a detailed neuropsychological battery administered by qualified research personnel based on the recommendation of the National Institute of Neurological Disorders and Stroke and the Canadian Stroke Network.^22^ The comprehensive neuropsychological assessment covers six cognitive domains:

1. Executive Function: Colour trails test A&B^23^
2. Attention Function: digit span forward and backward^24^
3. Language Function: 15-item modified Boston Naming Test^25^
4. Visuomotor speed: Symbol Digit Modalities Test^26^
5. Visuospatial Function: Rey Complex Figure Test-copy^27^
6. Memory Function: Rey Complex Figure Test-immediate/delayed recall and recognition (visual memory), and Hopkins Verbal Learning Test-immediate/delayed test recall and recognition (verbal memory)^28^

The cognitive scores across all domains were normally distributed, as confirmed by non-significant Shapiro–Wilk test results. This neuropsychological battery and the procedure of *Z*-score standardization have been validated among Asian older adults.^29^

Individual raw test scores were converted to standardized *Z*-scores based on the means and SDs of the whole group. The score for each cognitive domain was computed by averaging the *Z*-scores of individual tests and standardized using the composite mean and SD of the whole sample. To obtain the global cognition score for each participant, the domain-based *Z*-scores were averaged and standardized using the mean and SD of the whole sample. Additionally, we performed the five-minute Montreal Cognitive Assessment to quickly screen dementia in the community, which has good validity, reliability, and sensitivity in our prior studies.^30,31^

### MRI protocol and data analysis

Magnetic Resonance Imaging was acquired on a MAGNETOM Prisma 3T scanner using a 64 channel receiver head coil (Siemens Healthcare, Erlangen, Germany). Foam padding was placed around the subject’s head to make them comfortable and minimize head motion. All participants underwent T1 weighted magnetization-prepared rapid gradient echo (T1 MPRAGE) and fluid-attenuated inversion recovery (FLAIR) sequences. TRUST MRI was used to acquire OEF,^32^ Phase Contrast MRI was used to collect global CBF.^33^

### Structural MRI

Parameters for T1 MPRAGE included the following: field-of-view (FOV) = 256 mm, voxel size = 1.0 × 1.0 × 1.0 mm^3^, repetition time (TR) = 2.1 s, inversion time (TI) = 1.10 s, echo time (TE) = 3.8 ms, flip angle = 12 degrees; T2 FLAIR sequence parameters were described as follows: FOV = 220 mm, slice thickness = 5.0 mm, voxel size = 0.9 × 0.9 × 5.0 mm^3^, repetition time (TR) = 7.5 s, inversion time (TI) = 2.50 s, echo time (TE) = 91.0 ms, flip angle = 150 degrees.

T1 MPRAGE examination was acquired for brain volume quantification by using an automatic processing tool, MRICloud (https://www.MRICloud.org; Johns Hopkins University, Baltimore, MD). The imaging assessments of WMH grade follow the STRIVE criteria, which include separate ratings for periventricular and deep white matter hyperintensities.^34,35^ The WMH volumes were segmented by the Lesion Segmentation Tool (LST) algorithm^36^ as implemented in the LST toolbox version (3.0.0) for Statistical Parametric Mapping (SPM) (www.statistical-modelling.de/lst.html). The process included automatic segmentation, classification and quantification based on FLAIR images. Every FLAIR sequence that had an abnormal total WMH volume was manually inspected to ensure that there were no visible discrepancies, and we also carefully excluded infarcts from the WMH volume as inadvertent inclusion of infarcts in ‘WMH volume’ may inflate associations.^37^ WMH volume was log-transformed for further analysis due to its skewed distribution.

### OEF and CBF

Sequence parameters of TRUST were: single slice, axial FOV = 220 mm, voxel size = 3.4 × 3.4 × 5.0 mm^3^, repetition time (TR) = 3.0 s, inversion time (TI) = 1.02 s, echo time (TE) = 3.9 ms, labelling slab thickness = 100 mm, gap = 22.5 mm, four effective TEs (0.44, 40, 80 and 160 ms) and total scan time = 1.2 min.

OEF was assessed by the TRUST technique, the imaging slice was positioned 20 mm higher than the confluence of sinus to sample pure venous blood in the superior sagittal sinus with an orientation orthogonal to the sinus axis. The labelling slab was placed superior to the imaging slice to cover the sagittal sinus inflow. For data processing, mono-exponential fitting of the TRUST MRI signal as a function of eTE yielded blood T2. Blood T2 was in turn converted to *Y*_v_ using a calibration plot.^38^ Based on Fick’s principle,^2^ when fully oxygenated arterial blood flows through the capillaries, the surrounding brain tissue will extract the oxygen, thus the brain OEF can be calculated as:


$$OEF=\frac{Ya-Yv}{Ya}\times 100\text{\%}$$

Here, *Y*_a_ (assumed as 98%^8^) is the arterial oxygenation, *Y*_v_ is the venous oxygenation defined as the fraction of oxyhaemoglobin in the venous blood. Specifically, haematocrit values were assumed to be 42% for males and 40% for females.^39^

Before the flow measurements, time-of-flight angiogram was performed to obtain the anatomical information of the feeding arteries of the brain. Imaging parameters of the angiogram were: TR = 20 ms, TE = 3.12 ms, flip angle = 18, FOV = 120 mm, voxel size = 0.2 × 0.2 × 1.5 mm^3^, number of slices = 36, one 60-mm saturation slab positioned above the imaging slab, and scan duration = 46 s. To evaluate global CBF, Phase Contrast MRI (gradient echo) was performed targeting the four major cerebral arteries, i.e. the left and right carotid arteries (LICA and RICA) and the left and right vertebral arteries (LVA and RVA). Regions of interest (ROIs) were manually delineated on complex difference magnitude images to identify these arteries. PC MRI parameters included: TR = 8.6 ms, TE = 4.0 ms, flip angle = 20, FOV = 200 mm, Slice thickness = 5.0 mm, voxel size = 0.5 × 0.5 × 5 mm^3^, Venc = 40 cm/s, scan duration = 13 s. Segmentation and negative-phase correction were performed by Y.Y., an MR specialist with four years of experience, in accordance with the phase-contrast MRI literature.^33^ Flow rates, expressed in millilitres per minute, were calculated by integrating velocity across the delineated ROIs. Subsequently, CBF was calculated by dividing the total blood flow by the total brain volume. All processing was performed using in-house MATLAB (MathWorks, Natick, MA) scripts. Details of these MRI techniques and processing steps can be found in Lin *et al*.^8^

### Vascular risk factors

Vascular risk factors were assessed from self-reported clinical interviews (hypertension, diabetes, hyperlipidaemia: present/absent), and questionnaires (smoking history: ever/never). BMI was calculated as weight (kg)/height^2^ (m^2^), and obesity was defined as BMI > 28.^40^ We constructed a composite vascular risk score following prior literature^7,41^ by summing the presence of hypertension, diabetes, hyperlipidaemia, ever smoking, and obesity, yielding a range of 0–5. This score entered the main models as a continuous covariate.

In the stratified analysis, participants were divided into two vascular risk factor groups: Low vascular risk factor group (values of 0, 1 or 2) and high vascular risk factor group (values of 3, 4 or 5).^7^

### APOE genotyping

The APOE4 carrier status was determined and coded as follows: individuals with one or two E4 alleles were coded as 1, and individuals with no E4 alleles were coded as 0. Laboratory personnel were blinded to participants’ cognitive status. Because blood sampling was optional, APOE data were available for 140 participants who consented.

### Covariates

Age and sex were included as covariates in models. Educational level was recorded for each participant and further adjusted for in analyses involving cognitive outcomes.

### Statistical analysis

Sample characteristics are presented as mean ± SD or number (%), characteristics of participants across the four OEF/CBF quadrants were compared using appropriate statistical tests. Continuous variables are presented as mean ± SD or median (IQR) according to distribution and were compared with one-way ANOVA or the Kruskal–Wallis test. Categorical variables are summarized as counts (percentages) and were compared with *χ*² or Fisher’s exact tests, as appropriate. Ordinal variables (e.g. education level, Fazekas scores) were analysed using non-parametric tests. All tests were two-sided with a significance level of *α* = 0.05.

To establish OEF CBF patterns, a four-quadrant analysis was used.^42^ Participants were categorized into four quadrants based on median splits of CBF and OEF: High OEF + High CBF (Q1), High OEF + Low CBF (Q2), Low OEF + Low CBF (Q3), Low OEF + High CBF (Q4), to better understand the state of the brain oxygen utilization and perfusion. This quadrant-based approach is conceptually similar to methods used in prior work to describe joint patterns.^43^ Our four-pattern categorization was designed as a descriptive summary of physiologically meaningful joint states and the configuration with high OEF and low CBF corresponds to the historically described misery perfusion phenotype.

Associations between OEF CBF patterns and brain outcomes were examined using generalized linear models. For WMH, log-transformed WMH volume was modelled as the dependent variable, adjusted for age and sex. For cognition, both global cognition and domain-specific measures (attention, executive function, language, visuomotor function, visuospatial function, and memory) were analysed as outcomes, adjusted for age, sex and educational level. Given the research gap, the ‘High OEF + Low CBF’ quadrant was chosen as the reference group.

To further investigate potential nonlinear associations between OEF, CBF and brain outcomes, we employed response surface analysis (RSA) using second-order polynomial regression:


$$Z=b_{0}+b_{1}X+b_{2}Y+b_{3}X^{2}+b_{4}XY+b_{5}Y^{2}+covariates$$

where *X* represents OEF, *Y* represents CBF and *Z* represents WMH volume or cognition residuals (WMH outcomes were adjusted for age and sex; cognitive outcomes were additionally adjusted for education level), this technique enables visualization and formal statistical testing of interactive patterns across two conceptual dimensions: by using mean-centred OEF (*X*) and CBF (*Y*), the balance axis (CBF = OEF), capturing coordinated OEF–CBF states, and the bias axis (CBF = –OEF + 2*μ*, with *μ* representing the sample mean OEF), reflecting OEF–CBF mismatch. Along each axis, we tested the slope and curvature to evaluate whether matched versus mismatched states were associated with more adverse brain outcomes. Predicted outcome values (WMH volume or cognition residuals) were computed across sampled points on the response surface and plotted along these axes. This approach follows the principles outlined in Humberg *et al*.,^19^ and has been adapted here for neurophysiological data modelling.^20^ Response surface analyses were conducted using the ‘rsm’ and ‘plotly’ package in R, which implements standard response surface methodology.^44^

In the supplementary sensitivity analyses, generalized linear models were used to examine the linear relationships between OEF CBF and WMH, cognition. To further assess these relationships, we performed stratified analyses across vascular risk factor subgroups and APOE 4 subgroups to evaluate heterogeneity of associations. In addition, MANCOVA was applied to validate the RSA findings, with Pillai’s trace chosen as the primary multivariate test statistic. To identify specific domains driving the overall effect, follow-up ANCOVAs were conducted for each cognitive outcome. All analyses were conducted in RStudio (version 4.3.3).

## Results

### Sample characteristics

A total of 296 participants with complete OEF and CBF data were included in the study. Participants’ characteristics are detailed in Table 1. In the overall cohort, the mean age of the cohort was 67.8 ± 8.8 years, and 31.1% were male. The prevalence of hypertension, hyperlipidaemia and diabetes mellitus was 45.3%, 34.8% and 20.3%, respectively, and 20.6% reported a history of smoking. The mean BMI was 24.2 ± 3.2 kg/m^2^. The overall mean OEF was 39.7 ± 4.5%, and the mean global CBF was 58.2 ± 9.0 ml/100 g/min. The average Fazekas scores for deep matter was 1.29 ± 0.584, and the mean WMH volume was 3.38 ± 6.16. We also assessed participants’ cognitive status using the 5-min MoCA. The mean score in our cohort was 8.29 ± 2.54, indicating generally preserved cognitive performance. Participants across the four OEF CBF patterns showed generally similar characteristics, with differences mainly in sex distribution, smoking history, WMH volume and the expected OEF/CBF values by design.

**Table 1:** Characteristics of participants

	Overall (*n* = 296)	High OEF + High CBF (*n* = 54)	High OEF + Low CBF (*n* = 94)	Low OEF + Low CBF (*n* = 52)	Low OEF + High CBF (*n* = 96)	*P* value
Age (years)	67.8 ± 8.76	67.4 ± 8.86	69.2 ± 9.27	68.6 ± 8.84	66.1 ± 7.96	*P* = 0.16
Sex (male)	92 (31.1%)	9 (16.7%)	43 (45.7%)	29 (55.8%)	11 (11.5%)	***P*** **<** **0.001**
Education level						*P* = 0.44
Never went to school	48 (16.2%)	11 (20.4%)	17 (18.1%)	5 (9.6%)	15 (15.6%)	
Primary school	103 (34.8%)	18 (33.3%)	33 (35.1%)	22 (42.3%)	30 (31.3%)	
Middle school	65 (22.0%)	14 (25.9%)	21 (22.3%)	13 (25.0%)	17 (17.7%)	
High school/technical secondary school	44 (14.9%)	6 (11.1%)	15 (16.0%)	6 (11.5%)	17 (17.7%)	
College and above	36 (12.2%)	5 (5.6%)	8 (8.5%)	6 (11.5%)	17 (17.7%)	
Hypertension	134 (45.3%)	31 (57.4%)	45 (47.9%)	19 (36.5%)	39 (40.6%)	*P* = 0.12
Hyperlipidaemia	103 (34.8%)	23 (42.6%)	31 (33.0%)	19 (36.5%)	30 (31.3%)	*P* = 0.58
Diabetes mellitus	60 (20.3%)	16 (29.6%)	19 (20.2%)	11 (21.2%)	14 (14.6%)	*P* = 0.20
Smoking history	61 (20.6%)	6 (11.1%)	31 (33.0%)	16 (30.8%)	8 (8.3%)	***P*** **<** **0.001**
Body mass index (kg/m^2^)	24.2 ± 3.21	23.9 ± 3.23	24.6 ± 3.13	24.2 ± 2.77	23.8 ± 3.49	*P* = 0.40
APOE 4 allele carriers*	24(17.1%)	9(28.1%)	6(15.0%)	2(7.7%)	7(16.7%)	*P* = 0.22
Cerebral oxygen extraction fraction (%)	39.7 ± 4.45	42.0 ± 2.27	43.9 ± 3.23	36.2 ± 2.45	36.1 ± 2.27	***P*** **<** **0.001**
Global CBF (ml/100 g/min)	58.2 ± 8.95	64.2 ± 4.64	50.2 ± 5.35	52.5 ± 6.27	65.8 ± 5.31	***P*** **<** **0.001**
Global cognition *Z*-scores	0.00 ± 1.00	−0.08 ± 1.23	−0.13 ± 1.04	0.20 ± 0.78	0.11 ± 0.92	*P* = 0.18
5 min Moca Scores	8.29 ± 2.54	8.28 ± 2.93	8.08 ± 2.58	8.73 ± 2.30	8.28 ± 2.46	*P* = 0.51
Deep WMH Fazekas score	1.29 ± 0.584	1.28 ± 0.564	1.34 ± 0.597	1.33 ± 0.585	1.22 ± 0.584	*P* = 0.51
WMH volume (cm^3^) *	3.38 ± 6.16	2.74 ± 3.90	4.48 ± 8.02	4.33 ± 6.73	2.26 ± 4.57	***P*** **=** **0.025**

Bold values indicate statistically significant differences between groups (*P* < 0.05). *Participants with APOE genotype data (overall *n* = 140; by group *n* = 32, 40, 26, 42); WMH volume analyses excluded participants with infarcts (*n* = 254).

In the reference group (Q2: ‘High OEF + Low CBF’, *n* = 94), the mean age was 69.2 ± 9.3 years, with 45.7% being male. The prevalence of hypertension, hyperlipidaemia and diabetes mellitus was 47.9%, 33.0% and 20.2%, respectively, while 33.0% reported a history of smoking. The mean BMI was 24.6 ± 3.1 kg/m². APOE alle 4 carriers accounted for 15.0% of this group. By design, the mean OEF was relatively high (43.9 ± 3.2%), whereas global CBF was relatively low (50.2 ± 5.4 ml/100 g/min). The global cognition was −0.13 ± 1.04, lower compared with other groups. The average deep WMH Fazekas scores were 1.34 ± 0.60, and the mean WMH volume was 4.48 ± 8.02.

### Result of OEF–CBF pattern comparisons

**Figure 1:**
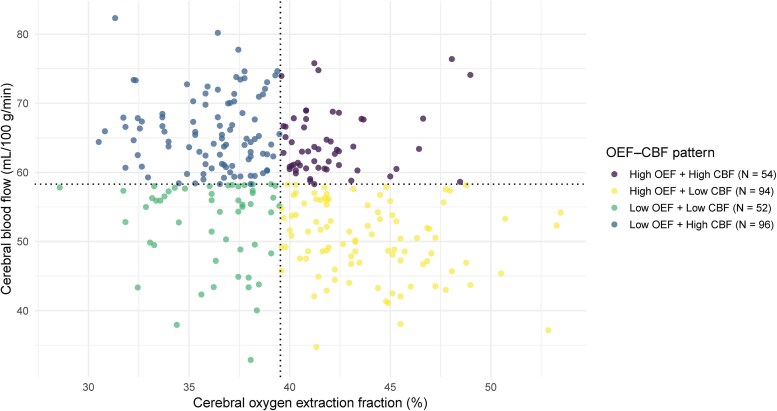
**Cerebral oxygen extraction fraction (OEF) and cerebral blood flow (CBF) quadrant classification.** Each dot represents one individual participant’s paired cerebral OEF (%) and CBF (ml/100 g/min) values. Colours correspond to the four OEF CBF patterns: High OEF + High CBF (*n* = 54), High OEF + Low CBF (*n* = 94), Low OEF + Low CBF (*n* = 52), and Low OEF + High CBF (*n* = 96).

The four quadrants defined by CBF and OEF are shown in Fig. 1, we were using the high OEF + low CBF group (‘misery perfusion’) as the reference, after adjusting for age, sex and vascular risk factors, individuals in Q4 (Low OEF & High CBF, *β* = −0.55, 95% CI = [−1.05, −0.05]) exhibited significantly lower WMH volume compared to those in High OEF & Low CBF, see Table 2.

**Table 2:** Comparison of cerebral OEF and CBF patterns in relation to log-transformed WMH volume

	Model 1	Model 2
High OEF + High CBF (Q1)	−0.52 (−1.11, 0.07)	−0.54 (−1.13, 0.04)
High OEF + Low CBF (Q2)	Ref	Ref
Low OEF + Low CBF (Q3)	−0.27(−0.89, 0.35)	−0.14(−0.76, 0.47)
Low OEF + High CBF (Q4)	**−0.56(−1.07, −0.05)**	**−0.55(−1.05, −0.05)**

OEF and CBF patterns were treated as exposures, with white matter hyperintensity volume as the outcome, and the High OEF + Low CBF group (Q2) served as the reference category. Model 1 was adjusted for age and sex, and Model 2 was additionally adjusted for vascular risk factors, with significant differences highlighted in bold to aid interpretation.

Regarding cognitive function, both global cognition and several cognitive domains showed significantly better performance in participants with lower OEF. After adjustment for age, sex, education and vascular risk factors, global cognitive scores were significantly higher in both Q3 (*β* = 0.32, 95% CI = [0.02, 0.61]) and Q4 group (*β* = 0.28, 95% CI = [0.02, 0.54]) compared to Q2 group (Table 3). Moreover, as details shown in Table 3, in the domain-based neurofunctions, the attention, visuospatial function and memory function showed similar results, after adjustment for age, sex, educational level and vascular risk factors, that participants with lower OEF had better abilities than High OEF and Low CBF group, Low OEF and Low CBF(Q3) was associated with higher attention (*β* = 0.38, 95% CI = [0.05, 0.70]), visuospatial (*β* = 0.33, 95% CI = [0.03, 0.63]) and memory function (*β* = 0.33, 95% CI = [0.03, 0.63]) compared with reference group. Similarly, Low OEF and High CBF was associated with better attention (*β* = 0.31, 95% CI = [0.03, 0.59]) and visuospatial function (*β* = 0.32, 95% CI = [0.06, 0.58]) compared with reference group.

**Table 3:** Comparison of cerebral OEF and CBF patterns in cognitive performance

		Model 1	Model 2
Global cognition	High OEF + High CBF (Q1)	0.02(−0.26, 0.30)	0.19 (−0.11, 0.49)
	High OEF + Low CBF (Q2)	Ref	Ref
	Low OEF + Low CBF (Q3)	**0.31(0.01, 0.61)**	**0.32** (**0.02, 0.61)**
	Low OEF + High CBF (Q4)	**0.28**(**0.02, 0.53)**	**0.28** (**0.02, 0.54)**
Executive function	High OEF + High CBF (Q1)	0.08(−0.26, 0.42)	0.07 (−0.28, 0.41)
	High OEF + Low CBF (Q2)	Ref	Ref
	Low OEF + Low CBF (Q3)	0.22(−0.10, 0.56)	0.21 (−0.12, 0.55)
	Low OEF + High CBF (Q4)	0.14(−0.15, 0.43)	0.09 (−0.20, 0.38)
Attention function	High OEF + High CBF (Q1)	0.08(−0.23, 0.40)	0.22 (−0.10, 0.55)
	High OEF + Low CBF (Q2)	Ref	Ref
	Low OEF + Low CBF (Q3)	**0.39**(**0.06, 0.71)**	**0.38** (**0.05, 0.70)**
	Low OEF + High CBF (Q4)	**0.31**(**0.03, 0.59)**	**0.31** (**0.03, 0.59)**
Language function	High OEF + High CBF (Q1)	0.05(−0.29, 0.38)	0.17 (−0.18, 0.51)
	High OEF + Low CBF (Q2)	Ref	Ref
	Low OEF + Low CBF (Q3)	0.15(−0.20, 0.50)	0.19 (−0.16, 0.53)
	Low OEF + High CBF (Q4)	0.24(−0.06, 0.54)	0.23 (−0.06, 0.53)
Visuomotor speed	High OEF + High CBF (Q1)	0.03(−0.26, 0.32)	0.14 (−0.15, 0.44)
	High OEF + Low CBF (Q2)	Ref	Ref
	Low OEF + Low CBF (Q3)	0.13(−0.16, 0.43)	0.13 (−0.17, 0.42)
	Low OEF + High CBF (Q4)	0.08(−0.17, 0.33)	0.09 (−0.17, 0.34)
Visuospatial function	High OEF + High CBF (Q1)	0.04(−0.26, 0.34)	0.23 (−0.07, 0.53)
	High OEF + Low CBF (Q2)	Ref	Ref
	Low OEF + Low CBF (Q3)	**0.35**(**0.05, 0.65)**	**0.33** (**0.03, 0.63)**
	Low OEF + High CBF (Q4)	**0.33**(**0.07, 0.59)**	**0.32** (**0.06, 0.58)**
Memory function	High OEF + High CBF (Q1)	0.03(−0.26, 0.31)	0.08 (−0.22, 0.38)
	High OEF + Low CBF (Q2)	Ref	Ref
	Low OEF + Low CBF (Q3)	**0.36**(**0.08, 0.64)**	**0.33** (**0.03, 0.63)**
	Low OEF + High CBF (Q4)	0.05(−0.20, 0.30)	0.13 (−0.12, 0.39)

OEF and CBF patterns were treated as exposures, and the High OEF + Low CBF group (Q2) served as the reference category. Outcomes include global cognition and six cognitive domains: executive function, attention, language, visuomotor speed, visuospatial function, and memory.

Model 1 adjusted for age, sex and education level, Model 2 additionally adjusted for vascular risk factors, with significant differences highlighted in bold to aid interpretation.

### The association between OEF, CBF and WMH

Having established group differences across OEF CBF patterns, we next examined OEF and CBF as continuous variables to test their associations with WMH burden. In univariate analyses, OEF showed no significant linear relationship with WMH volume (*P* = 0.324, Supplementary Fig. 1), whereas lower CBF was significantly associated with greater WMH burden (*P* = 0.036, Supplementary Fig. 2). We then stratified by vascular risk (low versus high; Supplementary Figs. 3 and 4) and observed a negative association between CBF and log-transformed WMH volume in the low-risk subgroup (*β* = −0.027, 95% CI = [−0.052, −0.002], Supplementary Fig. 4), while OEF was not significantly associated with WMH volume in either vascular risk group (Supplementary Fig. 3). Similarly, in analyses stratified by APOE genotype, we did not observe significant associations of either CBF or OEF with WMH volume in any APOE group (Supplementary Figs. 5 and 6).

**Figure 2:**
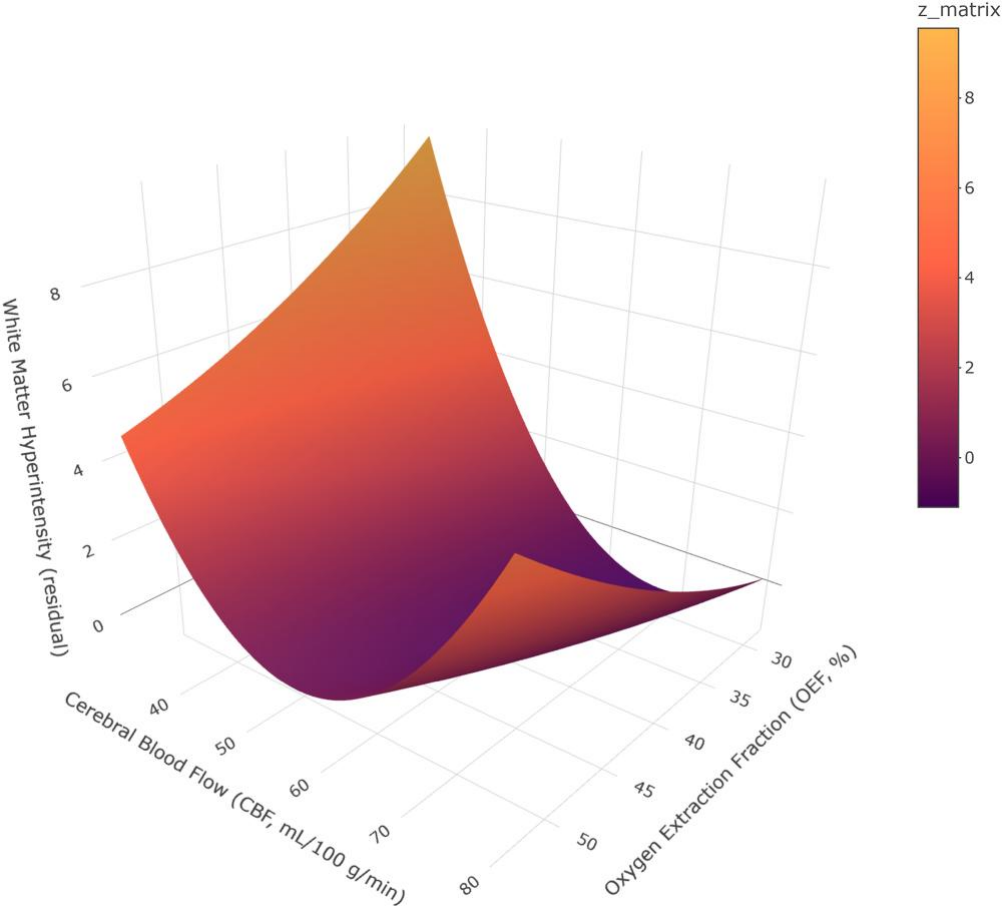
**A 3D surface map that predicts the relationship between OEF, CBF and WMH.** This 3D surface plot depicts the modelled relationship between OEF (%; *x*-axis), CBF (ml/100 g/min; *y*-axis) and residual WMH volume (cm^3^; *z*-axis). The surface shows predicted WMH volume residuals, with colour indicating the magnitude of the modelled response, and the colour bar labelled ‘*z*_matrix’ corresponds to the same modelled residual values, with warmer colours indicating higher residual WMH volume. Statistical analysis was performed using a response surface analysis with a sample size of *n* = 254 participants, and the model was adjusted for age and sex.

Nonlinear associations were further assessed using response surface analysis (Table 4). CBF showed a significant negative linear association with WMH residuals (CBF: *β* = −1.42, 95% CI = [−2.85, −0.01]), indicating that lower cerebral perfusion was associated with increased WMH burden, see Table 4. A significant positive quadratic effect of CBF was also observed (CBF²: *β* = 0.01, 95% CI = [0.00, 0.02]), indicating a curvilinear relationship. As shown in Fig. 2, WMH burden was elevated in most haemodynamic patterns (high OEF + high CBF, high OEF + low CBF, and low OEF + low CBF), whereas the low OEF + high CBF group exhibited relatively lower WMH volume. By contrast, coefficients along the bias and balance axes were not statistically significant, suggesting that CBF exerted a direct effect on WMH (Table 4).

**Table 4:** Coefficients of the response surface model for WMH volume and cognitive performance

	WMH outcome	Global cognition
OEF (*b*_1_)	−0.86(−3.92, 2.19)	**−0.49(−0.87, −0.11)**
CBF (*b*_2_)	**−1.42**(**−2.85, −0.01)**	−0.03(−0.20, 0.14)
OEF^2^ (*b*_3_)	0.00(−0.03, 0.03)	**0.01**(**0.00, 0.01)**
OEF × CBF (*b*_4_)	0.01(−0.01, 0.03)	0.00(−0.00, 0.00)
CBF^2^ (*b*_5_)	**0.01**(**0.00, 0.02)**	−0.00 (−0.00, 0.00)
Slope of Balance axis (*b*_1_ + *b*_2_)	−2.28(−6.28, 1.71)	**−0.52**(**−1.01, −0.04)**
Curvature along the Balance axis (*b*_3_ + *b*_4_ + *b*_5_)	0.02(−0.02, 0.02)	**0.01**(**0.00, 0.01)**
Slope of Bias axis (*b*_1_ − *b*_2_)	0.56(−1.99, 3.12)	**−0.46**(**−0.79, −0.13)**
Curvature along the Bias axis (*b*_3_ − *b*_4_ + *b*_5_)	0.00(−0.00, 0.03)	0.00(−0.00, 0.01)

Cerebral OEF, CBF, WMH.

$Z=b_{0}+b_{1}X+b_{2}Y+b_{3}X^{2}+b_{4}XY+b_{5}Y^{2}$ is the equation, *X* represents OEF, *Y* represents CBF and *Z* represents WMH volume or cognition residuals. Coefficients of second-order polynomial terms (linear, interaction, quadratic) are shown in table, while only Global cognition shown the significant coefficients (95% CI excludes 0) of the RSA balance and bias axis. WMH outcome adjusted for age, sex; Global cognition adjusted for age, sex and educational level, with significant differences highlighted in bold to aid interpretation.

### The association between OEF, CBF and cognition performance

We next examined whether OEF and CBF were associated with cognitive outcomes. In univariate analyses, higher OEF was significantly related to lower global cognition (*P* = 0.002), whereas CBF was not (*P* = 0.366, Supplementary Figs. 1 and 2). Extending this analysis to domain-specific functions, domain-specific analyses further confirmed consistent associations, with higher OEF linked to worse global cognition (*β* = −0.03, 95% CI = [−0.05, −0.01]), attention (*β* = −0.04, 95% CI = [−0.07, −0.02]), visuomotor (*β* = −0.04, 95% CI = [−0.06, −0.01]) and memory function (*β* = −0.03, 95% CI = [−0.05, −0.01]; Supplementary Table 1), after adjustment for age, sex, education level and vascular risk factors.

Furthermore, we stratified participants into low and high vascular risk groups; in the low vascular risk group, OEF was negatively associated with global cognition (*β* = −0.03, 95% CI = [−0.06, −0.01]) as well as attention, visuospatial and memory function, consistent with the findings in the overall sample (Supplementary Fig. 3). In APOE4 non-carriers, OEF was negatively associated with attention (*β* = −0.04, 95% CI = [−0.07, −0.00]) and visuospatial function (*β* = −0.05, 95% CI = [−0.08, −0.01]) but not with memory; however, in APOE4 carriers, OEF and visuospatial function showed a positive association trend (*β* = 0.10, 95% CI = [−0.01, 0.21]) that did not reach statistical significance (Supplementary Fig. 5). Together, these results indicate a robust inverse relationship between OEF and cognitive performance across both global and specific domains, most prominently in individuals with an overall low-risk profile (i.e. without APOE4 carriage or elevated vascular risk). By contrast, CBF in our cohort showed limited sensitivity in relation to cognitive performance (Supplementary Figs. 4 and 6).

**Figure 3:**
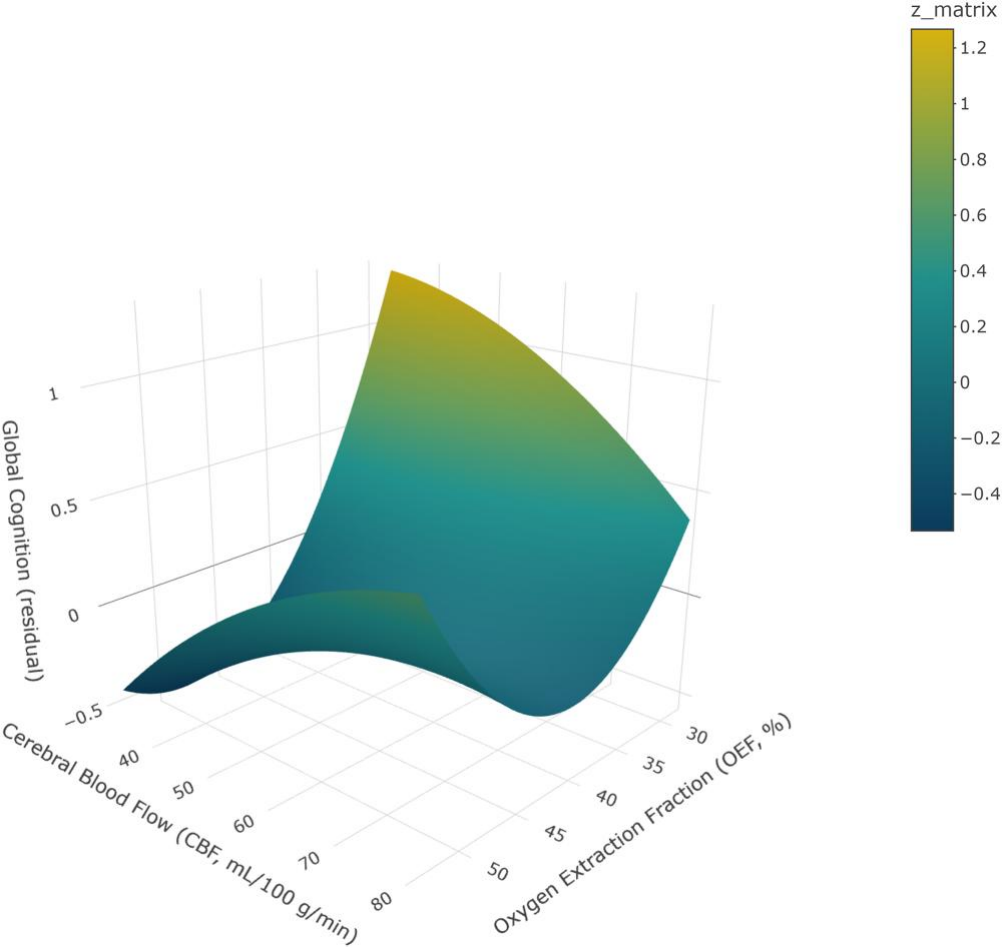
**A 3D surface map that predicts the relationship between OEF, cerebral blood flow and global cognition.** This 3D surface plot depicts the modelled relationship between OEF (%), CBF (ml/100 g/min), and residual global cognition. The surface shows predicted cognitive residuals (*z*-values), with colour indicating the magnitude of the modelled response, and the colour bar labelled ‘*z*_matrix’ corresponds to the same modelled residual values, with warmer colours indicating higher residual global cognition. Statistical analysis was performed using a response surface analysis with a sample size of *n* = 296 participants, and the model was adjusted for age, sex and educational level.

RSA was used to clarify the nonlinear relationships between the OEF, CBF and global cognitive performance. OEF showed a significant negative linear association with global cognition (*β* = −0.49, 95% CI = [−0.87, −0.11]), suggesting that higher oxygen extraction was related to poorer cognitive outcomes. Additionally, a significant positive quadratic effect of OEF was observed (OEF²: *β* = 0.01, 95% CI = [0.00, 0.01]), indicating a nonlinear (U-shaped) relationship and suggesting that matched changes in OEF and CBF influenced global cognition. Response surface analysis further illustrated these associations (Fig. 3). When CBF was relatively low, the association between OEF and cognition appeared largely linear, with higher OEF corresponding to poorer performance. At higher CBF levels, however, cognitive performance was relatively preserved even under higher OEF.

**Figure 4:**
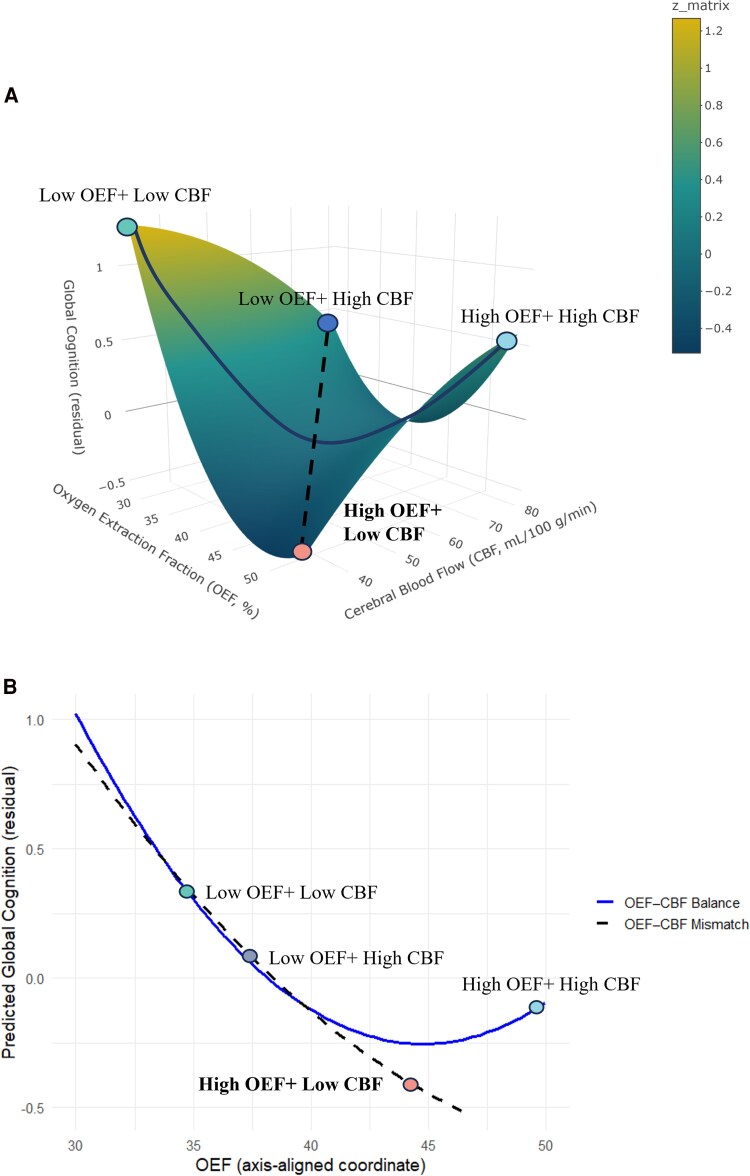
**Multidimensional modelling of OEF and CBF for predicting global cognition across model-derived balance and mismatch axe.** (**A**) This 3D surface plot illustrates the modelled relationship between OEF (%; *x*-axis), CBF (ml/100 g/min; *y*-axis), and residual global cognition (*z*-axis). Representative points are plotted to illustrate four extreme combinations of OEF and CBF (Low OEF + Low CBF, Low OEF + High CBF, High OEF + Low CBF, High OEF + High CBF). (**B**) This panel shows the predicted global cognition values along the OEF CBF balance axis (blue line) and the OEF CBF mismatch axis (black dashed line), derived from the same response surface model. The *x*-axis represents OEF expressed in an axis-aligned coordinate system, allowing visualization of cognitive differences along the balance and mismatch directions. The same four representative OEF CBF combinations are plotted for comparison. Higher values reflect better predicted cognitive performance. Statistical analysis is identical to that in panel A (*n* = 296 participants; model adjusted for age, sex and educational level).

Axis-based RSA was further performed to quantify systematic patterns along the balance and bias dimensions (Fig. 4). The *mismatch axis*, connecting the high-low and low-high corners, captures mismatched states. A significant linear effect was also observed along Mismatch line (*β* = −0.46, 95% CI = [−0.79, −0.13]), suggesting that increasing OEF in Mismatch line was generally associated with poorer cognitive performance. The *balance axis*, connecting the low OEF & low CBF and high OEF & high CBF corners, reflects matched OEF CBF states. Both the slope (*β* = −0.52, 95% CI = [−1.01, −0.04]) and the curvature (*β* = 0.01, 95% CI = [0.00, 0.01]) reached significance, indicating that matched changes in OEF and CBF exerted influence on the global cognition. As illustrated in Fig. 4, high OEF combined with high CBF was related to preserved cognition, a pattern consistent with the possibility that greater perfusion coincides with more favourable cognitive profiles in the context of elevated oxygen extraction.

### Sensitivity analysis

To evaluate the robustness of the OEF CBF and cognition associations beyond the linear and nonlinear terms already modelled in the RSA, we performed multivariate sensitivity analyses using MANCOVA. Consistent with the RSA findings, OEF showed a significant overall multivariate effect on cognition (Pillai’s trace, *F* = 2.84, *P* = 0.011), whereas CBF did not (*F* = 1.63, *P* = 0.139), and the OEF × CBF interaction was not significant (*F* = 0.34, *P* = 0.915; Supplementary Table 2). While MANCOVA corroborated a linear association between OEF and global cognition, RSA provided additional insights by revealing curvature and mismatch patterns that conventional linear models could not capture. In domain-specific analyses, higher OEF was associated with poorer executive (*F* = 4.51, *P* = 0.035), attention (*F* = 8.77, *P* = 0.003), visuospatial (*F* = 9.79, *P* = 0.001) and memory performance (*F* = 4.63, *P* = 0.032), whereas CBF was associated with memory function (*F* = 4.16, *P* = 0.042).

## Discussion

In this study, among community-based mid-life and senior adults, we examined associations of OEF and CBF with WMH burden and cognitive performance. CBF was negatively associated with WMH burden, whereas OEF was primarily and negatively associated with cognitive performance, particularly in attention, executive, visuospatial and memory domains. Individuals with high OEF combined with low CBF, the so-called ‘misery perfusion’ pattern showed the greatest WMH burden and the poorest cognition, identifying this as the high-risk subgroup, characterized by both greater WMH burden and poorer cognitive performance. Considering the nonlinear relationships in response surface analysis, higher OEF coupled with higher CBF was linked to relatively preserved cognition. Together, these findings highlight the importance of integrating oxygen utilization and perfusion measures for understanding cerebral small vessel disease and cognition, and they provide potential clinical implications for risk stratification.

### Associations of OEF and CBF with WMH burden

Previous studies have shown that OEF increases by approximately 0.14% per year with advancing age in older adults.^5,7^ Although WMHs are common in aging populations,^45^ the relationship between OEF and WMH remains inconsistent. From a global brain perspective, cross-sectional study reported no association,^3^ whereas longitudinal data suggested that increases in WMH volume may parallel increases in OEF.^7^ WMH, as a marker of chronic ischaemic stress, drives compensatory increases in OEF.^14,16^ Regionally, some studies reported elevated OEF in WMH regions,^17^ whereas others observed reduced OEF within lesion areas.^46^ In our study, we did not observe a significant positive linear association between OEF and logged WMH volume.

Instead, CBF plays a critical role in these cerebrovascular dynamics. Hypoperfusion and hypoxia are both key contributors to cerebrovascular disease, but their direct relationship is often difficult to disentangle due to the brain’s ability to compensate through autoregulatory mechanisms.^47^ The negative linear association between CBF and WMH in our findings aligns with established neuroimaging literature.^16^ Building on this, high OEF under low CBF characterizes the so-called misery perfusion state.^13^

Misery perfusion characterizes viable but at-risk neuronal tissue, where despite diminished CBF, metabolic demand persists, leading to elevated OEF.^48^ This mismatch state has also been associated with an increased risk of acute stroke.^13^ However, the mismatch between oxygen demand and availability may also be present in the absence of large vessel pathology and instead might originate from the capillary level.^49,50^ Our quadrant-based analysis confirmed that community individuals in the misery perfusion state (Q2, high OEF & low CBF) exhibited the greatest WMH burden, aligning with prior literature that OEF was higher in chronic ischaemia,^51^ and indicating a concurrent effect hypoperfusion and elevated oxygen utilization on white matter integrity.

By contrast, individuals with concurrently low OEF and low CBF may represent a hypometabolic or downregulated metabolic state, in which reduced oxygen utilization reflects adaptive suppression of metabolic demand or, at later stages, neurometabolic vascular failure commonly observed in Alzheimer’s disease.^14,43^ In our cohort, this quadrant showed relatively preserved cognition despite a greater WMH burden, suggesting that adaptive downregulation is the more parsimonious interpretation: chronic hypoperfusion may drive white-matter injury, whereas cognitive performance can be temporarily sustained through cognitive reserve. Consistent with this view, metabolic downregulation has been proposed as a potentially reversible, protective adaptation to sustained hypoperfusion.^52^

Within this physiological framework, consideration of the cerebral metabolic rate of oxygen (CMRO₂), the combination of CBF, OEF and arterial oxygen content, provides a more comprehensive view of brain energetic status.^53^ Although OEF often rises with aging to offset declining CBF and stabilize CMRO₂,^5,53^ higher OEF in the setting of low CBF does not signify favourable physiology^54^; rather, it reflects compensation under constraint and has been linked to limited cerebrovascular reserve, microvascular injury and progression of WMH.^55^ Conversely, when low CBF co-occurs with low OEF, the pattern likely indexes metabolic downregulation, potentially reversible in early stages,^52^ yet structural injury still accrues under chronic hypoperfusion. Taken together, lower perfusion is associated with higher WMH volume: whether accompanied by elevated OEF (constrained compensation) or by lowered OEF (demand downregulation),^52^ hypoperfusion remains the primary driver of white-matter damage and future risk.

### Associations of OEF and CBF with cognitive performance

Regarding cognitive performance, the relationship between OEF and cognition appears to be pathology-dependent. As Alzheimer’s disease progresses, reduced neural activity may lead to lower oxygen consumption,^56,57^ and OEF showed a progressive reduction from normal cognition to MCI and dementia, indicating a downward trend across groups.^3^ Especially in APOE 4 carriers, lower OEF related to worse executive functions^8^ and lower CMRO_2_ related to worse language and episodic memory performance.^57^ In our APOE 4 subgroup, OEF was positively associated with visuospatial function at a trend level, in line with previous literature.

In patients with vascular diseases, OEF tends to rise with vascular risk burden, consistent with disruption of structural and functional integrity of the cerebral vasculature, and with neurovascular uncoupling that compromises cerebral blood supply.^58^ Prior work shows stronger OEF and cognition associations in low-VRS strata,^3^ whereas such associations are attenuated in high-VRS groups. In our VRS-stratified analyses, participants with low vascular risk showed greater sensitivity of OEF to cognition, with OEF consistently and negatively associated with cognitive performance. In contrast, among those with high vascular risk, the OEF and cognition relationship was weaker and often non-significant, plausibly because vascular risk impairs cerebral autoregulation and neurovascular coupling, thereby obscuring the behavioural correlates of oxygen extraction.^15^

Notably, in aging sample, cognitively impaired individuals shown regionally elevated OEF compared with cognitively intact.^59^ Against this backdrop, in our full community-based cohort of older adults, higher OEF negatively associated with attention, visuospatial abilities and memory, showed consistent and sensitive associations. In contrast, several healthy aging cohorts without overt neurodegeneration, find no clear association between oxygen metabolism and cognitive measures,^53^ whereas CBF has consistently shown positive associations with cognitive performance,^60^ and even predicts longitudinal change,^61^ especially to attention/processing speed.^53^ In our cohort, similarly, higher CBF was specifically associated with better memory function.

When considering OEF and CBF jointly, the low-OEF/low-CBF and low-OEF/high-CBF groups showed better cognitive performance than the ‘misery perfusion’ group. The low-OEF/low-CBF pattern likely reflects metabolic downregulation, which reduces supply demand mismatch and mitigates immediate metabolic stress, permitting better cognition despite chronic hypoperfusion. The low-OEF/high-CBF pattern indicates adequate perfusion with modest demand, a state that is consistently associated with better cognition. Conversely, the high-OEF/low-CBF quadrant reflects compensation under constraint with exhausted reserve and microvascular injury, a profile repeatedly linked to poorer cognition and greater WMH burden. Our response surface analysis further indicated that the high OEF and high CBF were associated with relatively preserved cognition. Clinical evidence from carotid intervention trials suggests that augmenting flow may help preserve or improve cognition, supporting a causal role of perfusion in cognitive resilience.^62^ Sufficient perfusion therefore appears critical for sustaining performance, particularly in memory and attention domains.^53^

Last but not least, while cerebral metabolic dysregulation in AD stages often links lower metabolism to poorer cognition, our community cohort reflects the relatively healthy older adults in whom higher CMRO₂ is not uniformly advantageous across contexts. Rather, the combined OEF CBF pattern is most informative, indexing the states of supply and demand matching and microvascular efficiency. Mechanistically and clinically, improving perfusion can ease metabolic stress and support cognitive gains, underscoring the importance of hemodynamic stability in protecting brain health.^60^

### Clinical implications

Clinically, these findings underscore the potential of combined CBF and OEF assessments as markers for identifying individuals at risk for white matter deterioration and cognitive decline, even in the absence of overt clinical impairment. From a preventive standpoint, the mismatch pattern of high OEF and low CBF appears to play a critical role in brain vulnerability, our study suggests that timely detection and intervention during preclinical stages of cerebrovascular disease, particularly targeting haemodynamic stability, may mitigate future cognitive impairment and structural brain damage.^7^

### Strengths and limitations

This study's strength lies in its integration of innovative analytical techniques with a large community-based dataset. Despite these strengths, several limitations should be acknowledged. First, the WMH volume automatically identified by deep learning-based algorithms is still in development. As focal lesions and lacunes may inaccurately increase volume estimates due to incorrect identification, we excluded these participants. Second, phase contrast MRI provides global CBF measurements; regional assessments using Arterial Spin Labelling (ASL) in future studies may better capture lesion-specific perfusion, offering insights into personalized strategies. In addition, due to the cross-sectional design of the study, we cannot determine whether changes in CBF autoregulation precede OEF alterations or occur simultaneously, which should be tested in the future longitudinal study. Third, although we administered a comprehensive neuropsychological battery in this community-based cohort, cross-cohort comparability remains limited because we did not include widely harmonized global cognitive scales (e.g. full MoCA or MMSE). As a result, our community-based cohort of older adults, although free of diagnosed dementia, may include participants from normal cognition to early impairment, which reflects real-world community settings. Fourth, although we adjusted for several potential confounding factors, we acknowledge that the absence of direct Alzheimer’s disease biomarkers (e.g. amyloid or tau PET) limits our ability to fully exclude potential contamination from AD pathology. Moreover, to our knowledge, no universally accepted cross-modality clinical cutoffs exist for OEF or CBF in the context of chronic cerebrovascular disease. As such, our findings may not generalize to other ethnic groups, younger populations, or individuals with overt cerebrovascular disease. Broader applicability should be assessed in future studies that include diverse, clinically heterogeneous populations and leverage larger, multi-site cohorts with harmonized protocols.

## Conclusion

In summary, this community-based study demonstrates that higher OEF is associated with worse cognitive performance, and lower CBF is associated with higher WMH volume. The combination of high OEF and low CBF, a state of ‘misery perfusion’, was associated with the most adverse profile, marked by both greater WMH burden and poorer cognition. Importantly, when CBF was high, cognition was relatively preserved even under higher OEF, suggesting a buffering role of sufficient perfusion. These findings underscore the importance of considering oxygen utilization and perfusion both as biomarkers of cerebral small vessel disease and cognitive decline, and highlight potential opportunities for early risk stratification and preventive interventions targeting haemodynamic stability.

## Supplementary Material

fcag056_Supplementary_Data

## Data Availability

The data supporting the findings of this study are available upon reasonable request. Requests to access the datasets will be reviewed by corresponding authors Xin Xu at Zhejiang University to evaluate compliance with intellectual property protections and confidentiality agreements, contingent upon adherence to applicable Chinese laws and ethical regulations.
