# Supplementary material for: Cerebral oxygen extraction and blood flow in community-based older adults: associations with white matter hyperintensity and neurocognitive function
Source: Brain Commun. 2026 Feb 24;8(2):fcag056. doi: 10.1093/braincomms/fcag056 (PMC12971007; doi:10.1093/braincomms/fcag056)
Supplement: fcag056_Supplementary_Data [file fcag056_supplementary_data.pdf]

Supplementary Materials

Table Legend:

Supplementary Table 1. Association of Oxygen Extraction Fraction with Neurocognitive Function

Supplementary Table 2. Effects of Cerebral Blood Flow and Oxygen Extraction Fraction on Neurocognitive Function

Figure Legend:

Supplementary Figure 1. Association of **Oxygen Extraction Fraction** with White Matter Hyperintensity Volume and Global Cognition

Supplementary Figure 2. Association of **Cerebral Blood Flow** with White Matter Hyperintensity Volume and Global Cognition

Supplementary Figure 3. Association of **Oxygen Extraction Fraction** with Brain Outcomes Across Vascular Risk Groups

Supplementary Figure 4. Association of **Cerebral Blood Flow** with Brain Outcomes Across Vascular Risk Groups

Supplementary Figure 5. Association of **Oxygen Extraction Fraction** with Brain Outcomes Across APOE 4 Groups

Supplementary Figure 6. Association of **Cerebral Blood Flow** with Brain Outcomes Across APOE 4 Groups

Supplementary Table 1. Association of Oxygen Extraction Fraction with Neurocognitive Function

	Model 1	Model 2
Global Cognition	**-0.03(-0.06, -0.01)**	**-0.03(-0.05, -0.01)**
Executive function	-0.02(-0.05, 0.00)	-0.02(-0.05, 0.00)
Attention function	**-0.04(-0.07, -0.02)**	**-0.04(-0.07, -0.02)**
Language function	-0.01(-0.04, 0.01)	-0.01(-0.04, 0.01)
Visuomotor speed	-0.02(-0.04, 0.01)	-0.01(-0.04, 0.01)
Visuospatial function	**-0.04(-0.06, -0.02)**	**-0.04(-0.06, -0.01)**
Memory function	**-0.03(-0.04, -0.01)**	**-0.03(-0.05, -0.01)**

Note: cerebral oxygen extraction fraction was treated as exposure, and outcomes include global cognition and its six cognitive domains: executive function, attention, language, visuomotor speed, visuospatial function, and memory.

Model 1 is adjusted for age, sex and education level; Model 2 is additionally adjusted for vascular risk factors.

Supplementary Table 2. Effects of Cerebral Blood Flow and Oxygen Extraction Fraction on Neurocognitive Function

Global and domain-specific function	CBF		OEF		CBF×OEF	
	F	p-value	F	p-value	F	p-value
Global (MANCOVA, Pillai)	1.63	0.139	**2.84**	**0.011**	0.34	0.915
Executive function	2.88	0.091	**4.51**	**0.035**	0.61	0.437
Attention function	0.93	0.335	**8.77**	**0.003**	0.65	0.419
Language function	0.00	0.975	0.96	0.328	0.04	0.846
Visuomotor speed	2.31	0.130	1.23	0.269	0.09	0.752
Visuospatial function	0.18	0.700	**9.79**	**0.001**	0.10	0.744
Memory function	**4.16**	**0.042**	**4.63**	**0.032**	0.34	0.561

Note: MANCOVA tested the joint effect of cerebral blood flow and oxygen extraction fraction on global and all six cognitive domains simultaneously (Pillai's trace); ANCOVA results are presented for each cognitive domain separately. All models adjusted for age, sex, educational level and vascular risk factors.

Supplementary Figure 1. Association of **Oxygen Extraction Fraction** with White Matter Hyperintensity Volume and Global Cognition

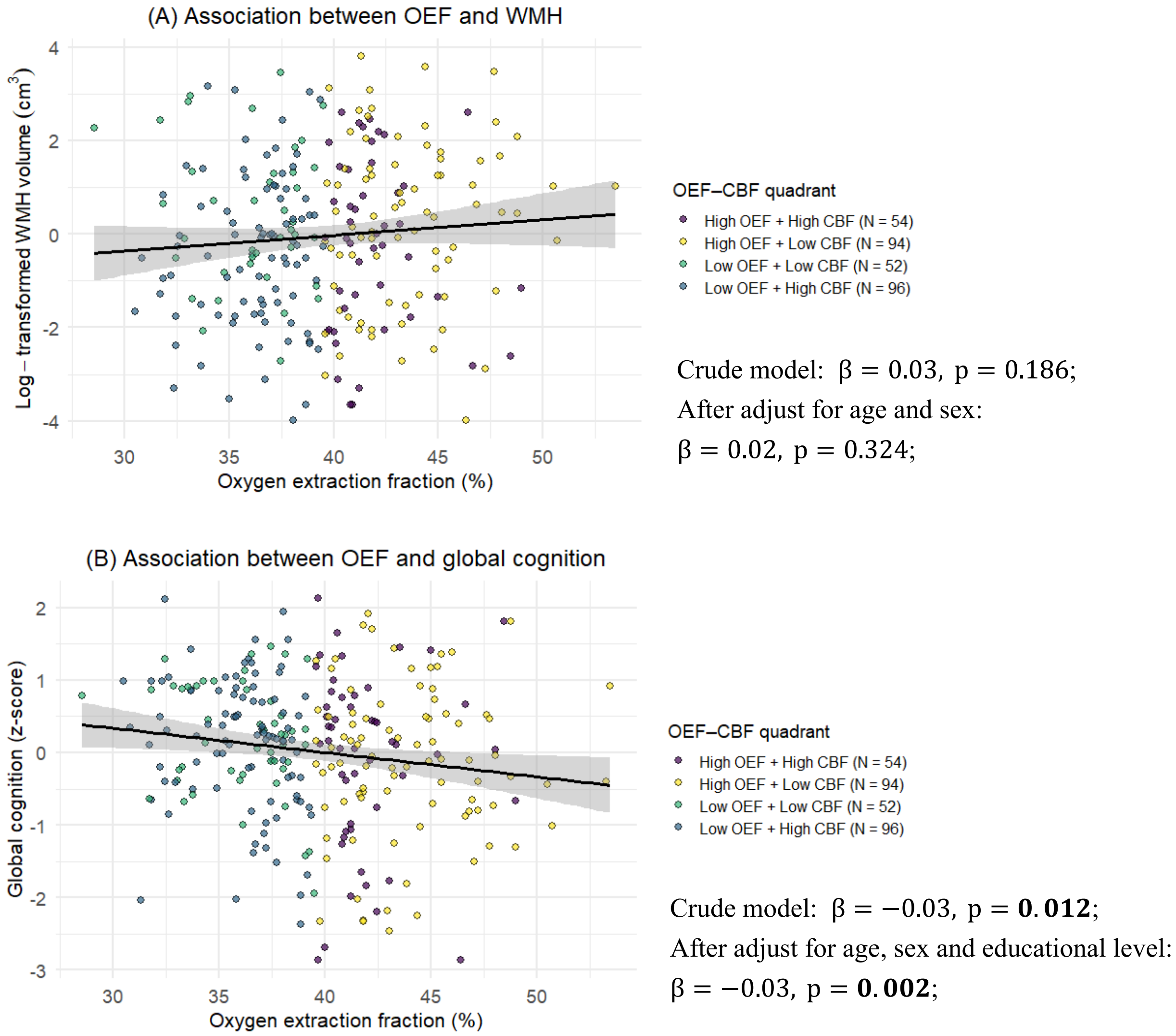

Note: OEF, cerebral oxygen extraction fraction (%); CBF, cerebral blood flow (ml/100g/min); WMH, white matter hyperintensity, expressed in log-transformed cubic centimeters (cm^3). Each point represents one participant, colour-coded by OEF CBF quadrant defined by median splits of OEF and CBF: high OEF + high CBF, high OEF + low CBF, low OEF + low CBF, and low OEF + high CBF. The corresponding sample sizes were n = 54, 94, 52, and 96, respectively. The solid black line represents the unadjusted linear fit with 95% confidence interval. All analyses were performed using a general linear model (GLM). **(A)** Association between oxygen extraction fraction and log-transformed WMH volume. The crude association was β= 0.03, p = 0.186; after adjustment for age and sex, the association attenuated (β= 0.02, p = 0.324). **(B)** Association between oxygen extraction fraction and global cognition. The crude association was β= -0.03, p = 0.012; after adjustment for age and sex, the association remained significant (β= -0.03, p = 0.002). Global cognition is expressed as a standardized z-score.

Supplementary Figure 2. Association of **Cerebral Blood Flow** with White Matter Hyperintensity Volume and Global Cognition

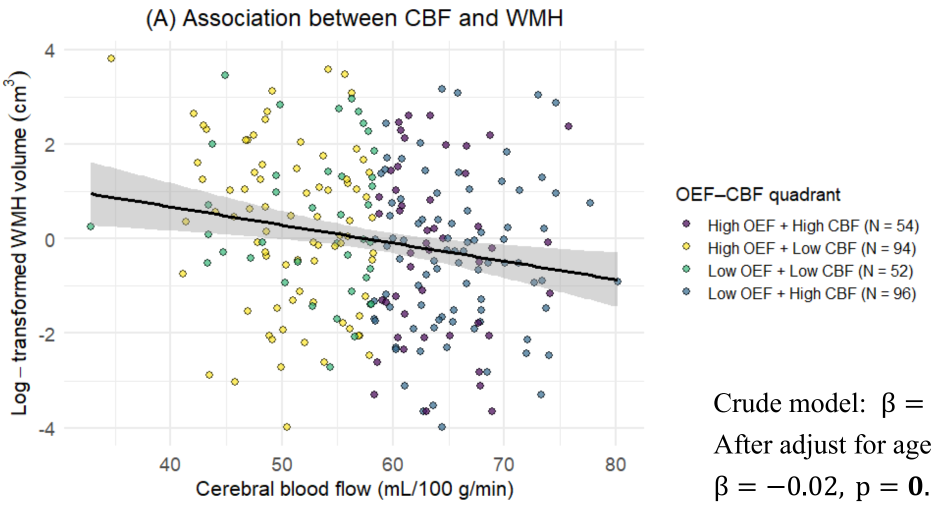

Crude model: $\beta = -0.04$, $p = \mathbf{0.001}$;
After adjust for age and sex:
$\beta = -0.02$, $p = \mathbf{0.036}$;

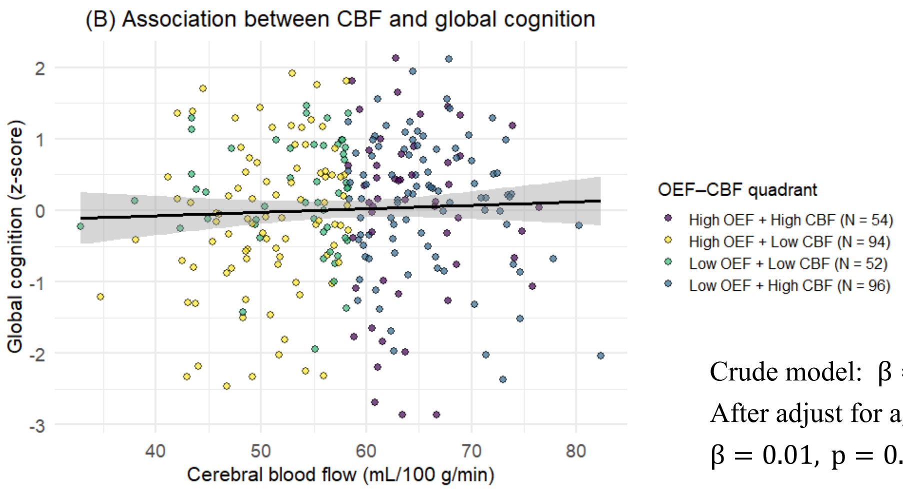

Crude model: $\beta = 0.01$, $p = 0.341$;
After adjust for age, sex and educational level:
$\beta = 0.01$, $p = 0.366$;

Note: OEF, cerebral oxygen extraction fraction (%); CBF, cerebral blood flow (ml/100g/min); WMH, white matter hyperintensity, expressed in log-transformed cubic centimeters (cm^3). Each point represents one participant, colour-coded by OEF CBF quadrant defined by median splits of OEF and CBF: high OEF + high CBF, high OEF + low CBF, low OEF + low CBF, and low OEF + high CBF. The corresponding sample sizes were n = 54, 94, 52, and 96, respectively. The solid black line represents the unadjusted linear fit with 95% confidence interval. All analyses were performed using a general linear model (GLM). **(A)**Association between cerebral blood flow and log-transformed WMH volume. In the crude model, higher CBF was associated with lower WMH volume (β = -0.04, p = 0.001); this association was attenuated but remained significant after adjustment for age and sex (β =-0.02, p = 0.036). **(B)** Association between cerebral blood flow and global cognition. The crude association was not significant (β = 0.01, p = 0.341) and remained non-significant after adjustment for age and sex (β = 0.01, p = 0.366). Global cognition is expressed as a standardized z-score.

Supplementary Figure 3. Association of **Oxygen Extraction Fraction** with Brain Outcomes Across Vascular Risk Groups

Outcome	Low vascular risk (N=250)	High vascular risk (N=46)
Logged WMH volume	0.013 (-0.034, 0.060)	0.081 (-0.038, 0.200)
Global cognition	-0.034 (-0.057, -0.011)	-0.027 (-0.088, 0.035)
Executive function	-0.017 (-0.043, 0.008)	-0.048 (-0.125, 0.030)
Attention function	-0.038 (-0.064, -0.012)	-0.063 (-0.127, 0.002)
Language function	-0.015 (-0.042, 0.012)	0.006 (-0.076, 0.087)
Visuomotor speed	-0.017 (-0.041, 0.007)	-0.008 (-0.071, 0.054)
Visuospatial function	-0.038 (-0.061, -0.014)	-0.020 (-0.081, 0.041)
Memory function	-0.035 (-0.061, -0.010)	-0.001 (-0.068, 0.066)

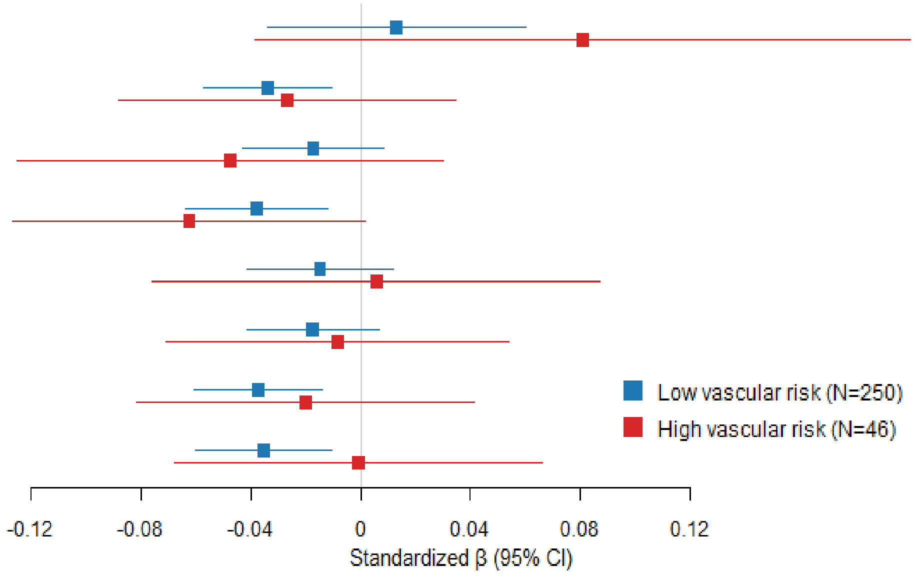

Note: Linear associations between cerebral oxygen extraction fraction and white matter hyperintensity burden and cognitive outcomes across vascular risk groups. OEF as the exposure. Standardized regression coefficients (β) and 95% confidence intervals are shown for participants with low vascular risk (N = 250) and high vascular risk (N = 46). Outcomes include log-transformed white matter hyperintensity (WMH) volume, global cognition and six cognitive domains: executive function, attention, language, visuomotor speed, visuospatial function, and memory. All analyses were performed using a general linear model (GLM). All models were adjusted for age, sex, and education (except the WMH model, which was adjusted for age and sex).

Supplementary Figure 4. Association of **Cerebral Blood Flow** with Brain Outcomes Across Vascular Risk Groups

**Outcome**	**Low vascular risk (N=250)**	**High vascular risk (N=46)**
Logged WMH volume	-0.027 (-0.052, -0.002)	0.019 (-0.053, 0.090)
Global cognition	0.007 (-0.006, 0.020)	-0.006 (-0.042, 0.029)
Executive function	0.003 (-0.012, 0.018)	-0.012 (-0.057, 0.033)
Attention function	0.007 (-0.007, 0.021)	0.004 (-0.035, 0.044)
Language function	0.009 (-0.006, 0.023)	-0.019 (-0.065, 0.027)
Visuomotor speed	0.002 (-0.011, 0.015)	-0.001 (-0.036, 0.035)
Visuospatial function	0.006 (-0.008, 0.019)	0.007 (-0.028, 0.042)
Memory function	0.006 (-0.008, 0.020)	0.005 (-0.033, 0.044)

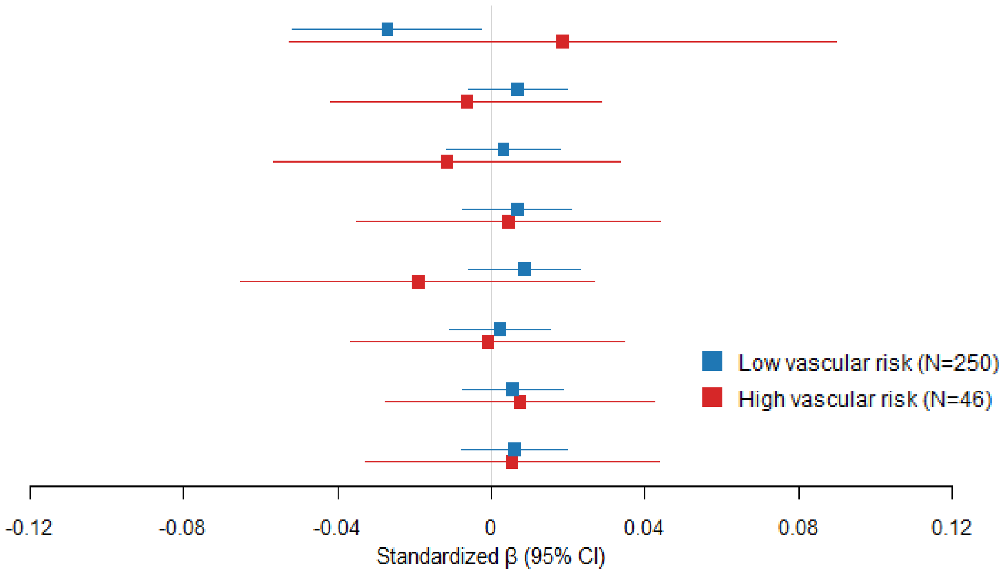

Note: Linear associations between cerebral blood flow and white matter hyperintensity burden and cognitive outcomes across vascular risk groups. CBF as the exposure. Standardized regression coefficients (β) and 95% confidence intervals are shown for participants with low vascular risk (N = 250) and high vascular risk (N = 46). Outcomes include log-transformed white matter hyperintensity (WMH) volume, global cognition and its six cognitive domains: executive function, attention, language, visuomotor speed, visuospatial function, and memory. All analyses were performed using a general linear model (GLM). All models were adjusted for age, sex, and education (except the WMH model, which was adjusted for age and sex).

Supplementary Figure 5. Association of **Oxygen Extraction Fraction** with Brain Outcomes Across APOE 4 Groups

**Outcome**	**APOE ε4 non-carriers (N=116)**	**APOE ε4 carriers (N=24)**
Logged WMH volume	-0.004 (-0.070, 0.062)	-0.050 (-0.305, 0.204)
Global cognition	-0.033 (-0.068, 0.002)	0.023 (-0.077, 0.122)
Executive function	-0.015 (-0.055, 0.025)	-0.090 (-0.227, 0.047)
Attention function	-0.037 (-0.072, -0.003)	-0.014 (-0.101, 0.073)
Language function	-0.019 (-0.065, 0.027)	0.033 (-0.081, 0.148)
Visuomotor speed	-0.017 (-0.053, 0.018)	0.024 (-0.079, 0.128)
Visuospatial function	-0.045 (-0.084, -0.005)	0.100 (-0.007, 0.207)
Memory function	-0.026 (-0.060, 0.008)	0.059 (-0.057, 0.176)

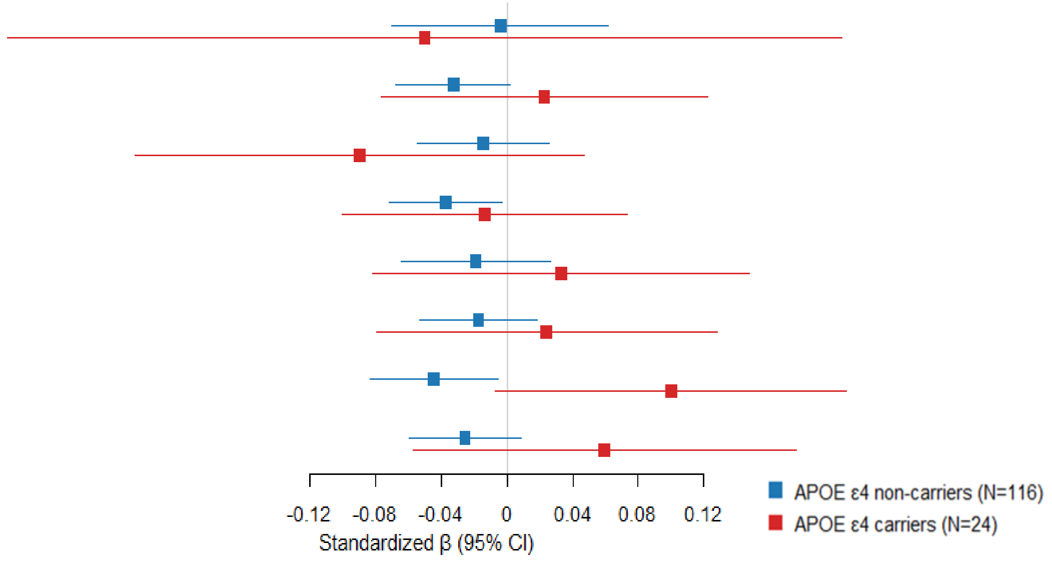

Note: Associations of oxygen extraction fraction (OEF) with white matter hyperintensity (WMH) volume and cognitive outcomes stratified by APOE ε4 carrier status. Standardized regression coefficients (β) and 95% confidence intervals are presented separately for APOE ε4 non-carriers (N = 116) and APOE ε4 carriers (N = 24). Outcomes include log-transformed WMH volume, global cognition and its six cognitive domains: executive function, attention, language, visuomotor speed, visuospatial function, and memory. All analyses were performed using a general linear model (GLM). All models were adjusted for age, sex, and education (except the WMH model, which was adjusted for age and sex).

Supplementary Figure 6. Association of **Cerebral Blood Flow** with Brain Outcomes Across APOE 4 Groups

Outcome	APOE ε4 non-carriers	APOE ε4 carriers
Logged WMH volume	-0.032 (-0.068, 0.004)	-0.007 (-0.113, 0.100)
Global cognition	0.002 (-0.017, 0.022)	0.027 (-0.033, 0.087)
Executive function	0.001 (-0.022, 0.025)	0.015 (-0.067, 0.097)
Attention function	-0.000 (-0.020, 0.019)	0.036 (-0.023, 0.096)
Language function	0.004 (-0.022, 0.029)	0.057 (-0.006, 0.121)
Visuomotor speed	0.001 (-0.018, 0.021)	0.026 (-0.035, 0.088)
Visuospatial function	0.003 (-0.019, 0.025)	0.003 (-0.067, 0.072)
Memory function	0.003 (-0.016, 0.022)	0.002 (-0.070, 0.074)

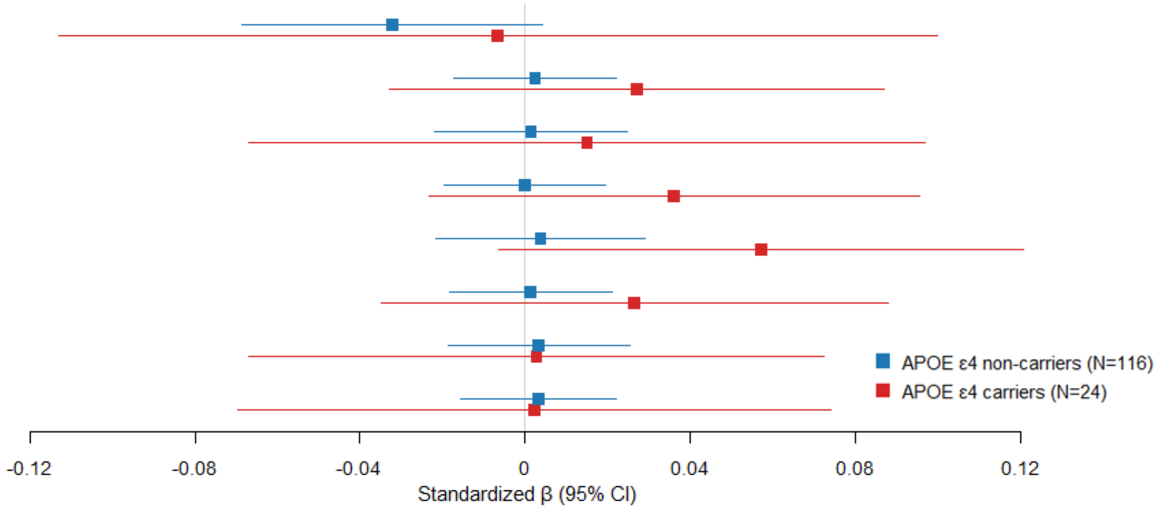

Note: Associations of cerebral blood flow (CBF) with white matter hyperintensity (WMH) volume and cognitive outcomes stratified by APOE ε4 carrier status. Standardized regression coefficients (β) and 95% confidence intervals are presented separately for APOE ε4 non-carriers (N = 116) and APOE ε4 carriers (N = 24). Outcomes include log-transformed WMH volume, global cognition and its six cognitive domains: executive function, attention, language, visuomotor speed, visuospatial function, and memory. All analyses were performed using a general linear model (GLM). All models were adjusted for age, sex, and education (except the WMH model, which was adjusted for age and sex).
